# Supplementary material for: Nanoimaging of the Edge-Dependent Optical Polarization Anisotropy of Black Phosphorus
Source: Nano Lett. 2022 Apr 5;22(8):3180–6. doi: 10.1021/acs.nanolett.1c03849 (PMC9052752; doi:10.1021/acs.nanolett.1c03849)
Supplement: Supplementary file 1 — nl1c03849_si_001.pdf [file nl1c03849_si_001.pdf]

Supporting Information for "Nano-imaging of the edge-dependent optical polarization anisotropy of black phosphorus"

Prakriti P. Joshi,[1] Ruiyu Li,[1,2] Joseph L. Spellberg,[1,2] Liangbo Liang,[3,a)] and Sarah B. King[1,2,b)]

[1)] *James Franck Institute, University of Chicago, Chicago, IL, 60637 USA*

[2)] *Department of Chemistry, University of Chicago, Chicago, IL, 60637 USA*

[3)] *Center for Nanophase Materials Sciences, Oak Ridge National Laboratory, Oak Ridge, TN, 37830 USA*

[a)] Electronic mail: liangl1@ornl.gov

[b)] Electronic mail: sbking@uchicago.edu

All referenced figures and tables can be found at the end of the Supporting Information.

METHODS

Sample Preparation and Characterization Few-layer black phosphorus (BP) is mechanically exfoliated from bulk crystals (Smart Elements) using a polydimethylsiloxane (PDMS) stamping method.[1] In brief, 2D BP is freshly cleaved from a bulk crystal with scotch tape, folded and peeled 3-4 times before it is transferred onto a piece of homemade PDMS stamp. The as-obtained PDMS is pressed onto a 380 μm-thick pre-cleaned Si chip with native oxide and gently peeled off from the surface, leaving thin BP flakes on the substrate. The flakes are first inspected using an optical microscope (Amscope, ME580TA-PZ-2L-18M3) with a 50x objective to estimate the thickness and morphology. After satisfying flakes are optically identified, the sample is mounted onto a PEEM sample plate with an optically accessible inner diameter of 5 mm. The aforementioned preparation is conducted in N_2 atmosphere with $O_2 < 1$ppm. After sample mounting the sample is removed from N_2 atmosphere, immediately transferred into the vacuum-chamber loadlock equipped with a fast-entry door, and pumped down to ultrahigh vacuum (UHV) conditions. The air exposure time during the whole transfer process is well controlled to under 20 seconds. After PEEM experiments are finished in UHV, the sample is removed from vacuum and stored in N_2 atmosphere again to prevent sample degradation before further characterization.

When removed from N_2 atmosphere for further characterization, an air-tight sample holder is used for transport to maintain inert atmosphere conditions. Raman microscopy is performed using a confocal optical microscope (Horiba LabRAM HR Evolution). BP is excited by a 532 nm laser and the scattered signal is collected using a 100x objective lens. Measurements take up to 20 minutes, during which the sample is exposed to ambient conditions. Tapping mode AFM (Bruker MultiMode 8) of the same flake is performed immediately after the Raman measurements.

Polarization-Dependent Photoemission Electron Microscopy Polarization-dependent photoemission electron microscopy (PEEM) measurements use laser illumination from an optical parametric chirped pulse amplifier (OPCPA) (Class 5 Photonics, 1 MHz) with central wavelengths of 800 nm (1.55 eV, FWHM 44 nm) and 515 nm (2.4 eV, FWHM 12 nm), and pulse durations $<$ 40 fs. The linearly polarized laser illumination is directed onto the BP sample in the PEEM chamber via a near-normal incidence Rh mirror, with an angle of incidence of 4°. Before entering the UHV chamber the laser light is transmitted through a thin-film polarizing beamsplitter (Eksma)

and rotated with an appropriate zero-order $\lambda/2$ waveplate (Thorlabs). We estimate fluences from the beam profile on the bare Si/native oxide substrate using the reported work function of n-doped Si, < 4.6 eV[2], meaning two-photon photoemission with 2.4 eV illumination and three-photon photoemission with 1.55 eV illumination. The fluence on the sample is $\sim$100 μJ/cm^2 for 2.4 eV illumination and $\sim$700 μJ/cm^2 for 1.55 illumination and is adjusted to limit space charging from the BP flake.

Annealing Black phosphorus flakes are annealed at 300°C and 350°C for 1 hour at each temperature and allowed to cool for $\sim$30 minutes before transferring to the PEEM. 350°C is the upper limit of the temperature range required to remove oxides and prepare a pristine black phosphorus surface while also avoiding degradation of black phosphorus.[3,4]

RH REFLECTIVITY CORRECTION

The PEEM sample is illuminated via pulsed laser that is reflected from a near-normal incidence (NNI) rhodium mirror inside the PEEM UHV chamber. The angle of incidence is 4° from normal, which means the electric field is effectively parallel to the sample plane. Therefore, polarization rotation of the laser illumination results in the rotation of the electric field in the sample plane. The reflectivity correction used here is adapted from Neff et al.[5] The effective intensity on the sample is dependent on the polarization-dependent reflectivity of the rhodium mirror, which can be described as the following:

$$I_{refl} = I_o[R_S \cos^2(\theta_{Rh}) + R_P \sin^2(\theta_{Rh})] \tag{1}$$

where I_o is the intensity of the illumination before the mirror (measured outside the chamber), R_S is the reflectivity of rhodium for s-polarized light, R_P is the reflectivity of rhodium for p-polarized light, and θ is the angle of polarization such that $\theta_{Rh} = 0°$ refers to s-polarization and $\theta_{Rh} = 90°$ refers to p-polarization with respect to the plane of incidence of the rhodium mirror.

For pump photon energy of 2.4 eV, we use a monolayer graphene/300 nm SiO_2/Si substrate to calibrate the reflectivity of the rhodium mirror. A representative image of the PEEM illumination on graphene/SiO_2/Si is shown in 4(a). We empirically determine the relative values of R_S and R_P and the angle $\theta_{Rh} = 0°$ by a power series (4(b)) and a polarization dependent measurement (4(c)) of a graphene/SiO_2/Si substrate. The work function of graphene is reported to be 4.6 eV.[6] We empirically determine the n-photon process of 2.4 eV with a power series. For a fixed polarization, $\theta_{Rh} = 0°$, we integrate the signal across the field of view for each power step and then

fit the resulting data with a fitting model $I_{PE,graphene} = Ax^b$ where x is the incident laser power, A is a coefficient, and b corresponds to the number of photons required for photoemission from graphene/SiO_2/Si (4(b)). We extract a value of 3.57 for b. For a given polarization, the intensity of the illumination reflected by the rhodium mirror, I_{refl}, is related to the photoemission intensity from graphene as $I_{refl} = \sqrt[m]{I_{PE,graphene}}$ such that $m = b$. In 4(c), we integrate across the entire field of view (FOV) and report the photoemission intensity as a function of polarization angle, θ. We fit the photoemission response with the fit $I_{PE} = A\cos^2(\theta - \beta) + C$. where A is the amplitude, β is the phaseshift, and C is the offset. For 515 nm, $\beta = 11.2°$. We take $\theta = \beta$, where the photoemission intensity is maximized, to be the angle at which the incident laser light is s-polarized with respect to the rhodium mirror, and the angle of minimum photoemission intensity $\beta + 90°$ to be the angle at which it is p-polarized.

To confirm the accuracy of our calibration, we extract a ratio of $\frac{R_S}{R_P}$ from our fit such that $\frac{R_S}{R_P} = \sqrt[m]{\frac{I_{PEfit,max}}{I_{PEfit,min}}}$. For m=b=3.57, $\frac{R_S}{R_P}$ = 1.20. The reported polarization-dependent reflectivity of rhodium is $R_{S,lit}$=0.82 and $R_{P,lit}$=0.68.[7] $\frac{R_{S,lit}}{R_{P,lit}}$=1.21, which means that our experimental calibration is in good agreement with known experimental values. The final polarization dependent photoemission signal from black phosphorus, $I_{PE,BP}(\theta)$, is from the photoemission signal, $I_{meas}(\theta)$, and the fit to the reflectivity of graphene: $I_{PE,BP}(\theta) = \frac{I_{meas}(\theta)}{[\sqrt[m]{I_{PE,graphene}(\theta)}]^n}$ where n is the number of photons required for photoemission from the black phosphorus sample and $I_{PE,graphene}(\theta)$ is normalized to 1. For a excitation photon energy of 2.4 eV, n = 2 (5(b)).

For excitation energy 1.55 eV, we generate a polarization-dependent calibration by using the literature values for the reflectivity of rhodium $R_{S,lit}$=0.81 and $R_{P,lit}$=0.76.[7] We perform a polarization-dependence on graphene/SiO_2/Si and extract $\beta = 11.8°$, which is consistent with the β extracted from 2.4 eV polarization dependence. The consistency of the phaseshift is to be expected, because that should be dependent on the orientation of the rhodium mirror inside the UHV chamber. Owing to a possible resonance state in graphene 3 eV below the Fermi level, we use the extracted β and previously reported R_S and R_P and model the reflectance of the rhodium mirror as:

$$I_{refl,1.55eV} = I_{refl} = I_o[R_S\cos^2(\theta_{Rh}) + R_P\sin^2(\theta_{Rh})] \tag{2}$$

where $\theta_{Rh} = \theta - \beta$. We normalize $I_{refl,1.55eV}$ to 1 and for each data set, we have $I_{PE,BP}(\theta) = \frac{I_{meas}(\theta)}{I^n_{refl,1.55eV}}$, where n = 3, as empirically determined by a power series on the black phosphorus flakes (5(e)).

ACROSS-BANDGAP POLARIZATION DEPENDENCE

In order to confirm that the polarization dependence is from the across-bandgap absorption and not the final state photoionization process, we perform a polarization dependence measurement with a 1.55 eV pump and a 3.1 eV probe such that the polarization of the pump is rotated from $\theta = 0^\circ$ to 400° in increments of 10°. With respect to time-zero, where the pump and probe pulses are temporally overlapped, negative time delays are where the 3.1 eV probe pulse arrives before the 1.55 eV pump pulse, and positive time delays are where the pump arrives before the probe. Because the work function of few-layer BP is > 4 eV,[8] the dominant photoemission process at negative time delays should be from the absorption of two 3.1 eV photons from the valence band or the absorption of one 3.1 eV photon and photoionization by a 1.55 eV photon. In contrast, the dominant photoemission process at positive time delays should be from the across bandgap absorption of a 1.55 eV photon and the subsequent photoionization of the intermediate state by a 3.1 eV photon. Another process, namely the absorption of two 1.55 eV photons and photoionization by a 3.1 eV photon is also possible, but less likely because it requires three photons instead of two. The power of the 3.1 eV illumination is 1.34 mW (333 kHz), and the 1.55 eV illumination is attenuated to 16.6 mW (333 kHz) such that there is no photoemission (3PPE) process from only 1.55 eV illumination. In this scheme, if the across bandgap absorption is indeed responsible for the observed polarization dependence, then the rotation of the polarization of the 1.55 eV pump should modulate the photoemission intensity only at positive time delays and not negative time delays, despite maintaining the same fluence of the pump and probe. In Fig. 13(a) and (b), we show the PEEM images at time delays of +0.475 ps and -0.475 ps, respectively, and in 13(c), we plot the integrated photoemission intensity from the body of the flake, which is marked in red in (a) and blue in (b). These time delays are chosen to avoid the cross-correlation of the pump and probe. We clearly observe a polarization modulation at +0.475 ps, but not at -0.475 ps, which confirms that the observed polarization modulation is not a polarization modulation of the final absorption. Because the β maps for 1.55 eV, a 3PPE process, and 2.4 eV, a 2PPE process, are extremely similar (Figs. 2 and 7), we can conclude that the polarization modulation is due to the across-bandgap absorption.

SUBLIMATION EXPERIMENTS

To probe the possibility of specific edge reconstructions contributing to the edge-dependent optical anisotropy, we heat a BP flake in UHV to the point of sublimation ($> 400°C$) to create eye-shaped holes with well-defined edges in the [100] (AC) direction.[3,9] 14(a) and (b) show PEEM images taken with broadband Hg-lamp (5.1 eV) illumination before and after sublimation. In addition to some sublimation at the edges of the flakes, we see obvious eye-shaped holes in the interior of the flakes with a depth of 1-3 nm, as confirmed by AFM in 14(c). Power series with $h\nu = 2.4$ eV before and after sublimation confirm a 2-photon photoemission process, as shown in figures 15 and 16. The polarization-dependence at $h\nu = 2.4$ eV shown for the unannealed and sublimated flake in figures 17 and 18, respectively. A comparison between the direction of the holes in 14(b) and 18(a) and the β values in 17(c) and 18(c) shows that the elongated holes are roughly aligned along $\theta = 110°$, providing secondary affirmation of our assignment to β as the AC direction. Close inspection of 17 and 18 show the appearance of holes in the ρ map of 18(d), but we do not observe any strong phaseshift around the hole edges in 18(c). Because the edges of the holes have a maximum depth of 3 nm on top of $\sim$100 nm (14(c) and (d)), we surmise that the absence of an apparent phaseshift is because of the small contribution of the edge signal compared to interior signal within the signal to noise ratio of our experiments. This observation is consistent with our DFT results discussed below and in the main text where the transitions from the interior are more intense than transitions at the edges, making it challenging to observe edge transitions with thin BP flakes.

ANGLE-DEPENDENT ELECTRONIC DIPOLE TRANSITIONS

For an optical transition from an electronic state i to another state f, the electronic transition dipole moment is given by $\mathbf{D} = \langle f|\nabla|i\rangle$, where the electronic wavefunctions $|i\rangle$ and $|f\rangle$ can be obtained by first-principles DFT calculations through the VASP package.[10] Then the transition dipole moment $\mathbf{D}$ is computed using the VASPKIT code.[11] It is typically a complex vector and can be defined as $\boldsymbol{D} = \begin{pmatrix} D_x \\ D_y \\ D_z \end{pmatrix} = \begin{pmatrix} |D_x|e^{i\phi_x} \\ |D_y|e^{i\phi_y} \\ |D_z|e^{i\phi_z} \end{pmatrix}$. When the electron-photon interaction is treated within the dipole approximation, the optical transition matrix element is proportional to $\boldsymbol{P} \cdot \boldsymbol{D}$, where $\boldsymbol{P}$ is the electric polarization vector of the light and defined as $\boldsymbol{P} = (\cos\theta, \sin\theta,\ 0)$. The optical

absorption intensity is then given by $I \propto |\boldsymbol{P} \cdot \boldsymbol{D}|^2$. To obtain a nonzero intensity, $\boldsymbol{D}$ should have a component parallel to the light polarization vector $\boldsymbol{P}$, which is an important selection rule for the optical transition.[10]

The relation between the intensity and light polarization angle can be shown as

$$
\begin{aligned}
I &\propto \left| (\cos\theta, \sin\theta,\ 0) \begin{pmatrix} |D_x| e^{i\phi_x} \\ |D_y| e^{i\phi_y} \\ |D_z| e^{i\phi_z} \end{pmatrix} \right|^2 \\
&\propto \left| (|D_x| \cos\theta e^{i\phi_x} + |D_y| \sin\theta e^{i\phi_y}) \right|^2 \\
&\propto (|D_x| \cos\theta e^{i\phi_x} + |D_y| \sin\theta e^{i\phi_y})\ (|D_x| \cos\theta e^{-i\phi_x} + |D_y| \sin\theta e^{-i\phi_y}) \\
&\propto |D_x|^2 \cos^2\theta + |D_y|^2 \sin^2\theta + |D_x||D_y| \cos\theta \sin\theta (e^{i(\phi_x - \phi_y)} + e^{-i(\phi_x - \phi_y)}) \\
&\propto |D_x|^2 \cos^2\theta + |D_y|^2 \sin^2\theta + 2|D_x||D_y| \cos\theta \sin\theta \cos(\phi_x - \phi_y) \\
&\propto |D_x|^2 \left[\cos^2\theta + \frac{|D_y|^2}{|D_x|^2} \sin^2\theta + 2\frac{|D_y|}{|D_x|} \cos\theta \sin\theta \cos(\phi_x - \phi_y) \right] \\
&\propto |D_x|^2 \left[\cos^2\theta + r^2 \sin^2\theta + 2r \cos\theta \sin\theta \cos\phi_{xy} \right] \qquad (3)
\end{aligned}
$$

The formula suggests that the maximum intensity direction depends on the ratio $r = \frac{|D_y|}{|D_x|}$ and the phase angle difference $\phi_{xy} = \phi_x - \phi_y$. To determine the angle corresponding to the maximum intensity, we need to do $\frac{\partial I}{\partial \theta} = 0$, which gives

$$
\tan 2\theta_{\max} = -\frac{2r\cos\phi_{xy}}{r^2 - 1}
$$

To understand the experimentally observed phase-shifted behavior at the edges of BP flakes, we carried out first-principles DFT calculations on a (1,3) reconstructed edge of monolayer BP that has been studied previously.[12] The armchair (AC) direction is defined as (1,0) while the zigzag (ZZ) direction corresponds to (0,1). Fig. 19(a) shows the calculated electronic band structure of the BP nanoribbon with the (1,3) edge, where the valence band maximum and conduction band minimum are denoted as VBM and CBM, respectively. By computing the contribution of the edge atoms to each band state (see the red circles), we can determine the two bands below the Fermi level (VBM and VBM-1) as edge bands. To determine whether different electronic transitions respond differently to the linear polarization of the excitation photons, we selected two representative transitions around 0.95 eV involving different valence and conduction states, as indicated by the arrows in Fig. 19(a). Note that the indices of transitions (#2 and #11) indicate the ordering of

these transitions among all possible transitions of 0.95 eV, as will be discussed in Table I below. For transition #2 in Fig. 19(b), the spatial distributions of charge densities of its initial valence state and final conduction state are plotted. The initial state is from an edge band (VBM-1) that has charge densities localized around the edges of the nanoribbon, while the final state has charge densities that are mostly localized around the edges with a certain degree of extension towards the interior. Similar charge density distributions are found for the initial and final states in transition #11 in Fig. 19(c), where the initial state is from another edge band (VBM). For both transitions, because the electronic wavefunctions and charge densities are confined at the rough edges of BP that have significant symmetry reduction compared to the interior atomic structure, their spatial distributions and symmetries are considerably modified compared to those in the interior of BP. Consequently, the transition dipole moments of both transitions are notably rotated away from the AC direction, i.e., the horizontal direction or 0.0° in Fig. 19. Such phase shifts are evident in the polarization-angle-dependent optical absorption profiles shown in Fig. 19(b-c), where the angle of maximum absorption is 47° and 69°, respectively. These results are in stark contrast to typical optical transitions that occur in the interior of BP. Taking the transition from VBM-2 to CBM at kpoint #2 as an example (Fig. 20(b)), both initial and final states correspond to typical ones from the interior of the nanoribbon (i.e., the bulk part), and thus the transition dipole moment and the maximum absorption are along the AC direction, an expected result for 2D BP systems, where the absorption coefficient for an electric field polarized along the armchair direction of the lattice significantly exceeds that of the zigzag direction when the excitation energy is below 3 eV.[10,13] In short, our calculations demonstrate that the optical selection rule is clearly changed at the edges due to the 1D confinement and symmetry reduction in comparison with the 2D BP, leading to orientation changes of the transition dipole moments at the edges and subsequently experimentally observed phase variations in the maximum absorption direction between the edges and interior. Similar modifications of optical selection rules due to edges have been observed in BP by Raman spectroscopy with the appearance of edge-dependent Raman modes.[14]

In addition to studying a few representative optical transitions in the (1,3)-edge nanoribbon, as described above (also in the main text), we considered all possible transitions between the valence bands and conduction bands in the Brillouin zone (BZ) that satisfy the energy conservation relation: $|\varepsilon_{c\mathbf{k}} - \varepsilon_{v\mathbf{k}} - \omega| \leq 0.03$ eV, where $\varepsilon_{c\mathbf{k}}$ ($\varepsilon_{v\mathbf{k}}$) indicates the energy of an conduction (valence) band state at the kpoint $\mathbf{k}$, ω corresponds to the incoming photon energy, and 0.03 eV represents the δ function. Please note that because of the limitations of energy accuracy with DFT, this dis-

cussion is qualitative rather than for quantiative comparison with photon energies discussed in the main text of the paper. For an optical transition of 0.95 eV, there are 13 eligible transitions in total, as shown in Table I. For each transition, we calculated the transition dipole moment and the polarization-angle-dependent optical absorption. The maximum intensity and the maximum absorption direction are shown in Table I, where different transitions clearly show different intensities and maximum absorption directions. Since all 13 transitions are related to edge states, their maximum absorption directions are all shifted away from the armchair direction. Finally, we computed the averaged angle-dependent intensity by considering contributions from all the transitions, following the formula

$$\bar{I}(\theta) = \frac{\sum_{i=1}^{N} I_i(\theta) W_i}{\sum_{i=1}^{N} W_i}, \tag{4}$$

where N is the total number of transitions (13 in this case), $I_i(\theta)$ is the intensity of transition i at the polarization angle of θ, and W_i represents the weight of transition i (set as 1 for equal contribution). As shown in Table I, the maximum absorption direction for the optical transition of 0.95 eV is at 22.0° after averaging (in other words, 22.0° phase shift away from the armchair direction). For an optical transition of 1.00 eV, the situation becomes more complicated, since it involves 24 eligible transitions between the valence and conduction bands at different k-points, as shown in Fig. 20 and Table II. More importantly, there are 4 transitions from VBM-2 to CBM (transition #1-#4) where both initial and final states correspond to the interior (or bulk) states (see Fig. 20(b) as an example), and therefore the optical selection rule is unaffected and the maximum absorption direction is along the AC direction (i.e., 0.0°) for these 4 transitions. For the other 20 transitions shown in Table II, their initial and/or final states are localized at the edges (Fig. 20(c-d)), and thus their maximum absorption directions are shifted away from the AC direction. We note that transitions #1-#4 originating from the interior show significantly stronger intensities than other transitions related to the edges, and hence they make dominant contributions to the averaged intensity shown in Eq. 4. Eventually, the averaged angle of maximum intensity is at 2.0° for the optical transition of 1.00 eV, as shown in Table II.

Finally, for the monolayer BP nanoribbon with the (1,3) edge, we calculated the averaged angle of maximum intensity as a function of the excitation photon energy in the range of 0.75-1.40 eV (Fig. 21). The averaged phase shift is mostly within ±10°. For excitation energies that do not involve transitions between the interior states (e.g., around 0.95 eV and around 1.20 eV), the phase shift is notably larger and can be more than 20°. Note that DFT calculations tend to underestimate

the electronic band gap of BP systems, and the calculated band gap of monolayer BP is less than 50% of the experimental optical gap.[12,13] Therefore, the excitation energies discussed in our calculations cannot be directly compared to the experimental values such as 1.55 and 2.40 eV. Nevertheless, the DFT results discussed above provide insightful information regarding optical transition processes and reveal the underlying microscopic picture of how the edges of BP modify the electronic wavefunctions and the optical selection rules, giving rise to edge-specific absorption profiles that are different from the absorption behaviors observed in 2D (or interior) BP flakes. Numerically, our calculations corroborated the experimental findings that the phase shifts with respect to the AC direction can be significant at the edges.

MEDIA

Please see attached videos for full background-corrected data sets of Flake 1 illuminated with 1.55 eV and 2.4 eV (Fig.s 1(c)-(e), 2, 6 - 8), Flake 2 illuminated with 2.4 eV (Figs. 3 and 9), and Flake 3 illuminated with 2.4 eV before and after annealing to 350° C (Fig. 10 and 11). They are named 'Figure2_Flake1_800nm.avi', 'Figure2_Flake1_515nm.avi', 'Figure3_Flake2_515nm.avi', 'Figure5_Flake3_unannealed.avi', and 'Figure5_Flake3_annealed.avi', respectively.

REFERENCES

[1] A. Castellanos-Gomez, M. Buscema, R. Molenaar, V. Singh, L. Janssen, H. S. J. v. d. Zant, and G. A. Steele, "Deterministic transfer of two-dimensional materials by all-dry viscoelastic stamping," 2D Materials **1**, 011002 (2014).

[2] A. Novikov, "Experimental measurement of work function in doped silicon surfaces," Solid-State Electron. **54**, 8–13 (2010).

[3] X. Liu, J. D. Wood, K.-S. Chen, E. Cho, and M. C. Hersam, "In Situ Thermal Decomposition of Exfoliated Two-Dimensional Black Phosphorus," J. Phys. Chem. Lett. **6**, 773–778 (2015), 1502.02644.

[4] A. Kumar, F. Telesio, S. Forti, A. Al-Temimy, C. Coletti, M. Serrano-Ruiz, M. Caporali, M. Peruzzini, F. Beltram, and S. Heun, "STM study of exfoliated few layer black phosphorus annealed in ultrahigh vacuum," 2D Materials **6**, 015005 (2018), 1808.09164.

[5]A. Neff, F. Niefind, B. Abel, S. C. B. Mannsfeld, and K. R. Siefermann, English"Imaging Nanoscale Morphology of Semiconducting Polymer Films with Photoemission Electron Microscopy," Adv. Mater. **29**, 1701012 (2017).

[6]Y.-J. Yu, Y. Zhao, S. Ryu, L. E. Brus, K. S. Kim, and P. Kim, "Tuning the Graphene Work Function by Electric Field Effect," Nano Lett. **9**, 3430–3434 (2009), 0909.0020.

[7]J. H. Weaver, C. G. Olson, and D. W. Lynch, "Optical investigation of the electronic structure of bulk Rh and Ir," Phys. Rev. B **15**, 4115–4118 (1977).

[8]Y. Cai, G. Zhang, and Y.-W. Zhang, English"Layer-dependent Band Alignment and Work Function of Few-Layer Phosphorene," Sci. Rep. **4**, 6677 (2014), 1409.8418.

[9]M. Fortin-DeschÃªnes, P. L. Levesque, R. Martel, and O. Moutanabbir, "Dynamics and Mechanisms of Exfoliated Black Phosphorus Sublimation," The Journal of Physical Chemistry Letters **7**, 1667–1674 (2016).

[10]X. Ling, S. Huang, E. H. Hasdeo, L. Liang, W. M. Parkin, Y. Tatsumi, A. R. T. Nugraha, A. A. Puretzky, P. M. Das, B. G. Sumpter, D. B. Geohegan, J. Kong, R. Saito, M. Drndic, V. Meunier, and M. S. Dresselhaus, "Anisotropic Electron-Photon and Electron-Phonon Interactions in Black Phosphorus," Nano Lett. **16**, 2260–2267 (2016).

[11]V. Wang, N. Xu, J.-C. Liu, G. Tang, and W.-T. Geng, "Vaspkit: A user-friendly interface facilitating high-throughput computing and analysis using vasp code," Computer Physics Communications **267**, 108033 (2021).

[12]L. Liang, J. Wang, W. Lin, B. G. Sumpter, V. Meunier, and M. Pan, "Electronic Bandgap and Edge Reconstruction in Phosphorene Materials," Nano Lett. **14**, 6400–6406 (2014).

[13]V. Tran, R. Soklaski, Y. Liang, and L. Yang, "Layer-controlled band gap and anisotropic excitons in few-layer black phosphorus," Phys. Rev. B **89**, 235319 (2014).

[14]H. B. Ribeiro, C. E. P. Villegas, D. A. Bahamon, D. Muraca, A. H. Castro Neto, E. A. T. de Souza, A. R. Rocha, M. A. Pimenta, and C. J. S. de Matos, "Edge phonons in black phosphorus," Nat. Commun. **7**, 12191 (2016), 1605.00032.

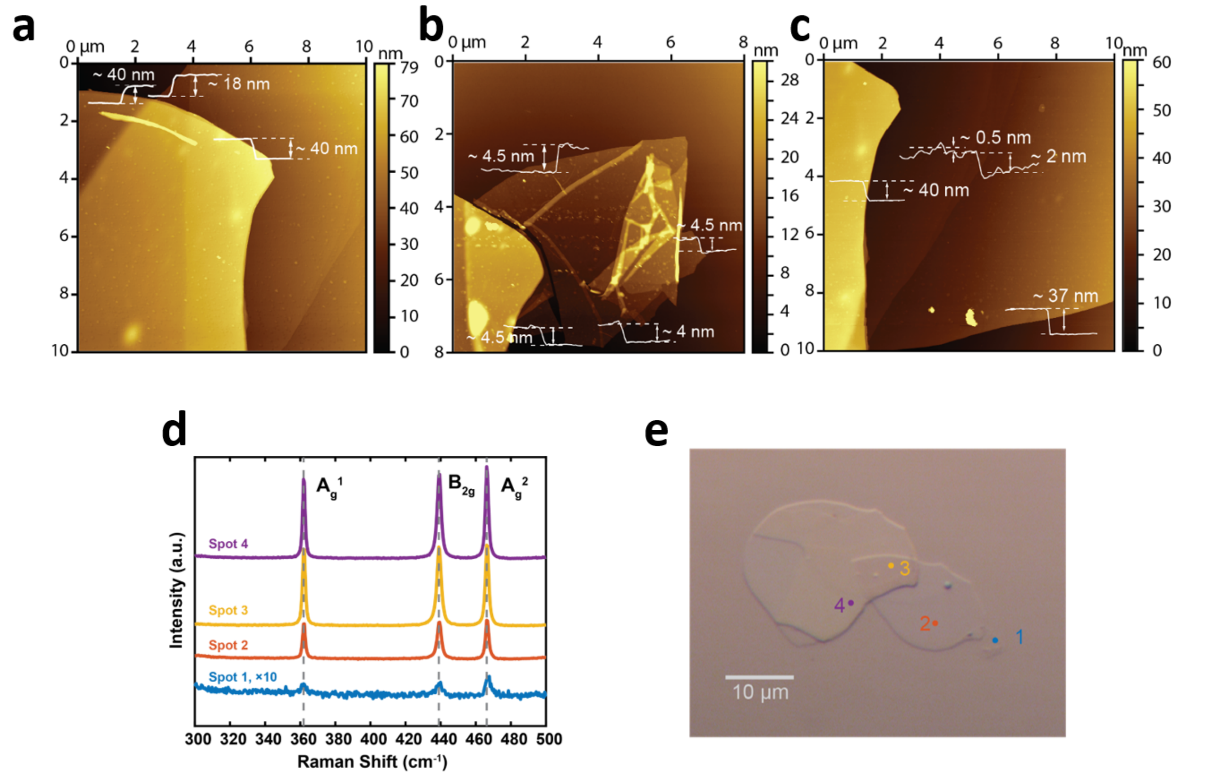

FIG. 1. Characterization of few layer BP flake 1. (a)-(c) AFM images of different portions of few-layer black phosphorus sample on Si with 2 nm native oxide layer. Thicknesses range from 4 nm (8 layers) to 58 nm (116 layers). (d) Raman spectroscopy of black phosphorus, with characteristic A_g^1, B_{2g}, and A_g^2 peaks at 362, 439, and 466 cm^{-1}, respectively. Raman spectra were taken from the marked spots on the optical image. (e) Optical image of BP flake on Si substrate with native oxide.

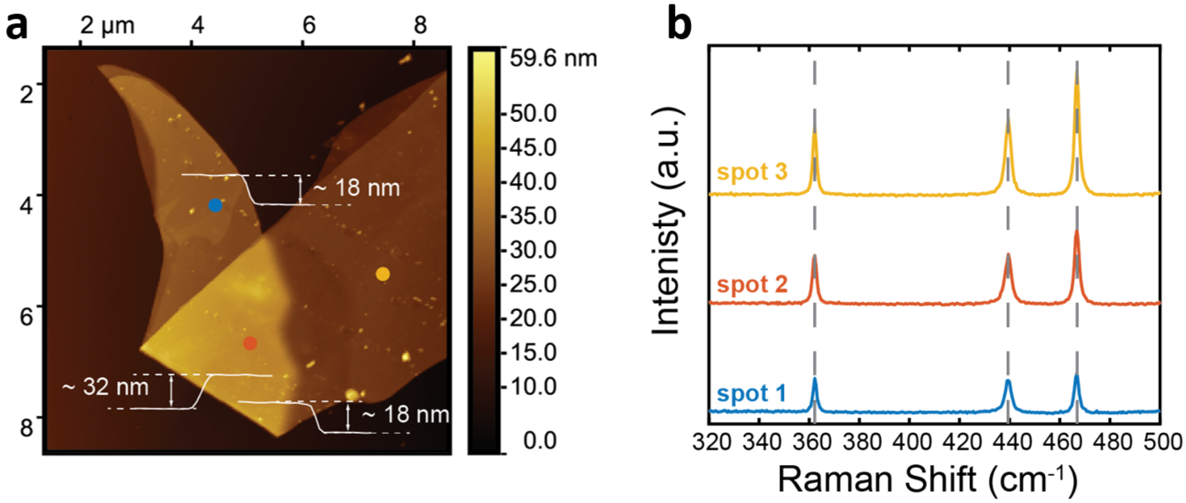

FIG. 2. Characterization of few layer BP flake 2. (a) AFM image of few-layer BP sample on Si with 2 nm native oxide layer. The upper left piece of the flake is folded under the main body of the flake. The non-overlapped regions of the flake are each ≈18 nm (36 layers) thick, and the region of overlap is measured to be 32 nm thick. The blue, red, and yellow points denote where Raman spectra were taken. (b) Raman spectroscopy of black phosphorus, with characteristic A_g^1, B_{2g}, and A_g^2 peaks at 362, 439, and 466 cm^{-1}, respectively.

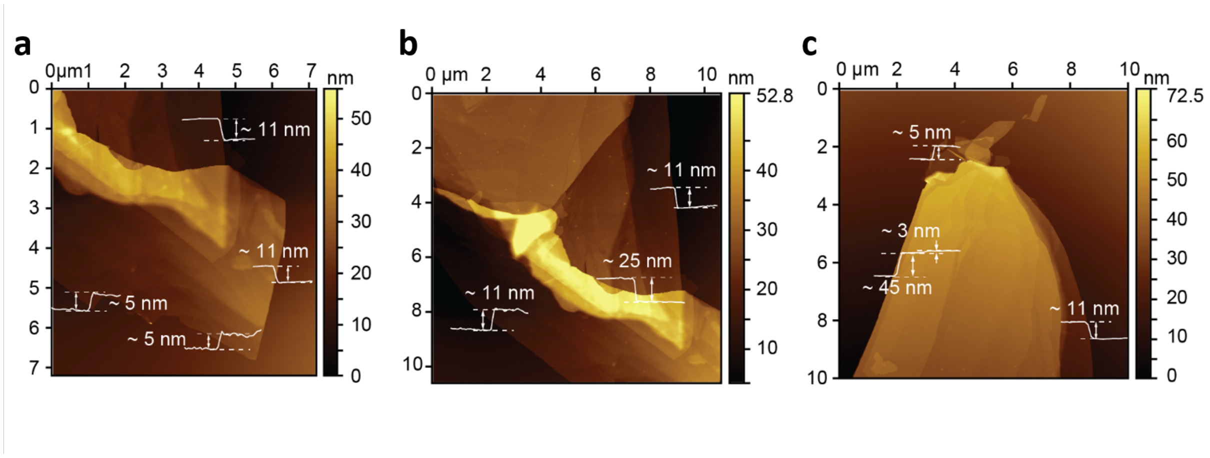

FIG. 3. Characterization of few layer BP flake 3. (a)-(c) AFM images of few-layer BP sample on Si with 2 nm native oxide layer. The measured thicknesses range from 5 nm (10 layers) to 45 nm (90 layers). In (a) and (b), a portion of the flake (11 nm thick) appears to have broken during mechanical exfoliation. A thicker portion (25 nm thick) appears to be the region in which these two broken pieces overlap. AFM images were taken post-annealing at 350°C.

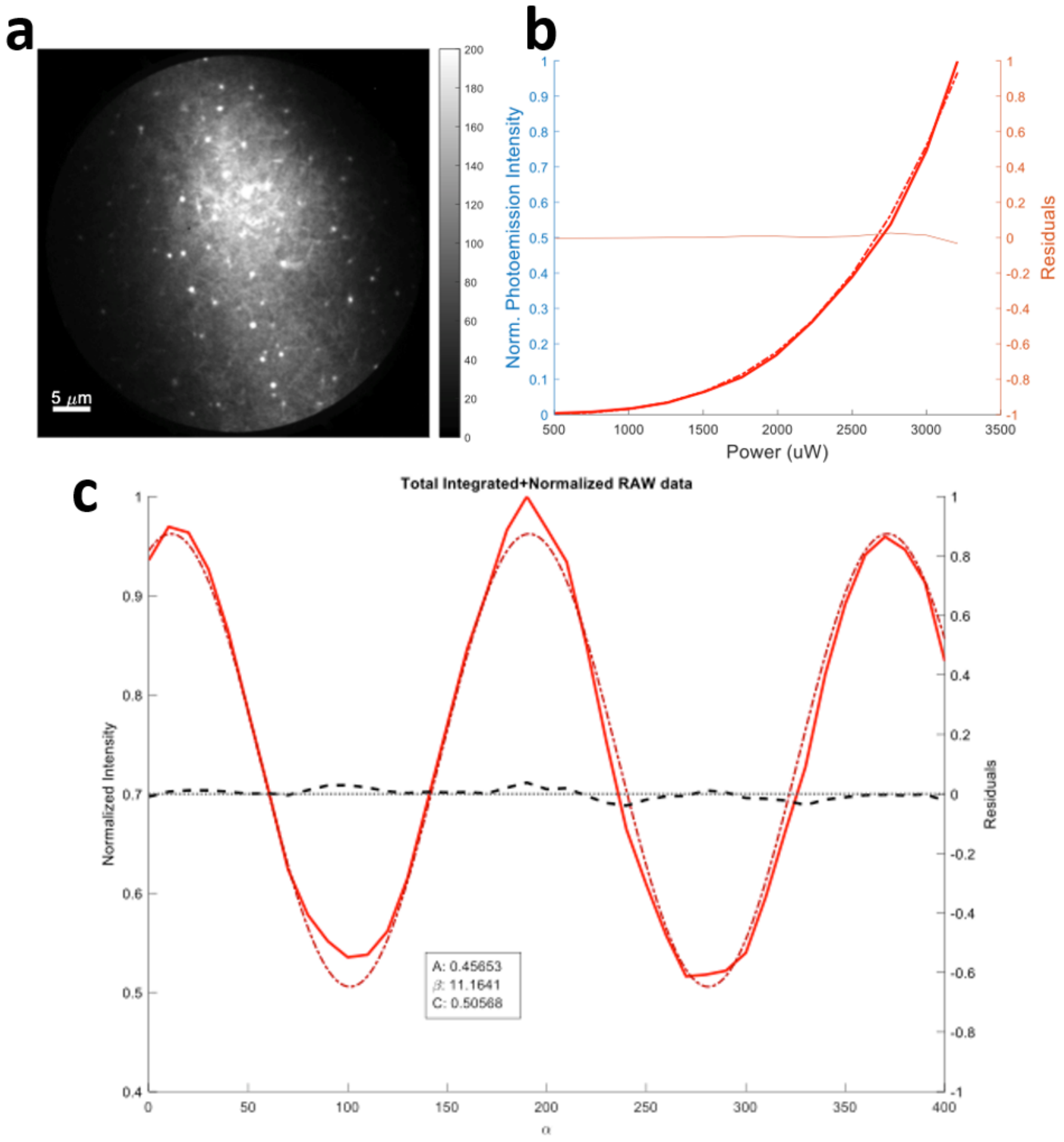

FIG. 4. Rh mirror calibration for 2.4 eV with graphene/SiO_2/Si substrate. (a) PEEM image with 2.4 eV illumination. Scale bar is 5 μm. (b) Power dependence of the integrated field of view (FOV) shown in (a). Power series is fit with the equation $I_{PE} = Ax^b$ where x is the incident laser power, A is a fitting coefficient, and b is the number of photons required for the photoemission process. The residuals of the fit are plotted against the right hand y-axis. (c) Polarization dependence with incident power of 3.21 mW (solid line) and fit with equation 1 (dashed line).

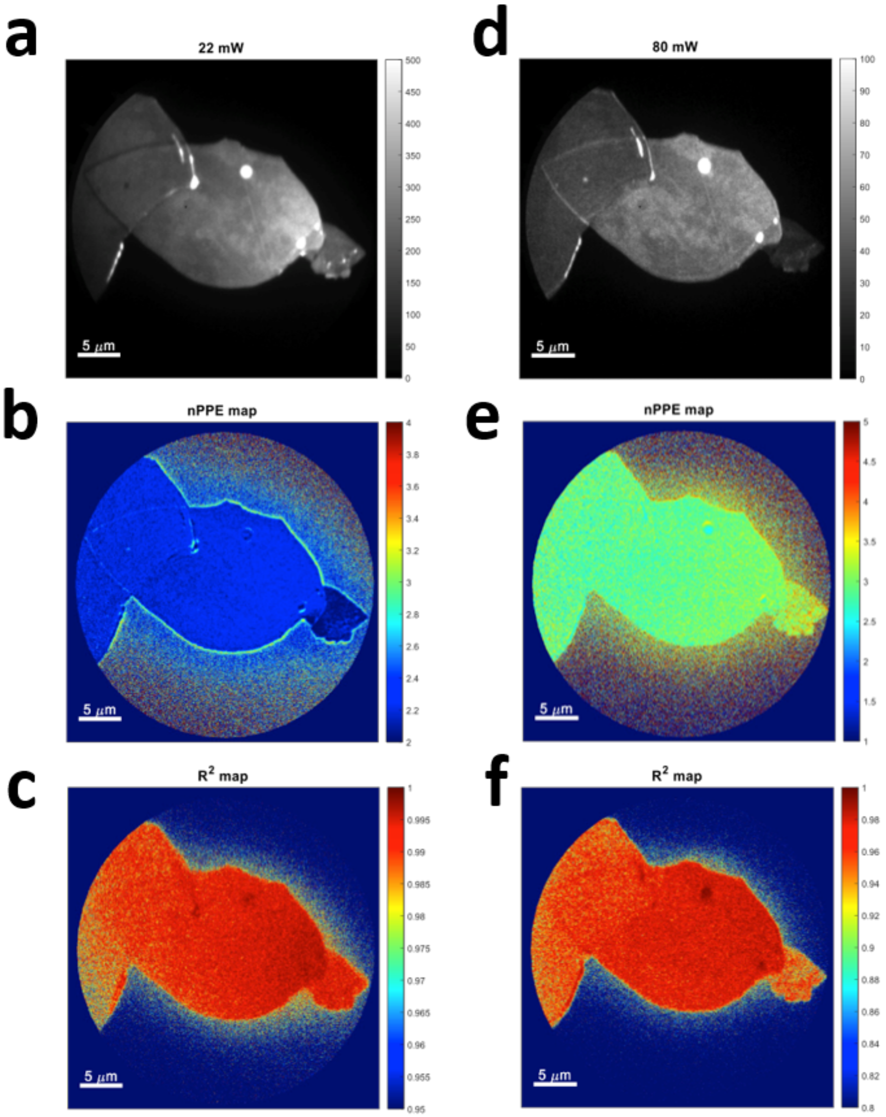

FIG. 5. Representative power series for 2.4 eV and 1.55 eV illumination. (a)-(c) Power series data for BP flake 1 with 2.4 eV. Data was taken with laser powers from 8 mW to 22 mW in 2 mW increments, staying under the space charging limit. (a) PEEM image taken with 22 mW. (b) Pixel-by-pixel map fit with equation $I_{PE,graphene} = Ax^b$ where x is the incident laser power, A is a fitting coefficient, and b is the number of photons required for the photoemission process. The value of b is shown for each pixel. For hν=2.4 eV, the photoemission is clearly a 2-photon process, as expected for few-layer BP. (c) The pixel-by-pixel map of goodness-of-fit parameter, R^2, as extracted from the fit described in (b). (d)-(f) Power series data with 1.55 eV. Data was taken with laser powers from 30 to 80 mW in 5 mW increments. (d) PEEM image taken with 80 mW. (e) Pixel-by-pixel map as described in (b). For hν=1.55 eV, the photoemission is clearly a 3-photon process, as expected for few-layer BP. (f) Pixel-by-pixel map of R^2 from fit in (e).

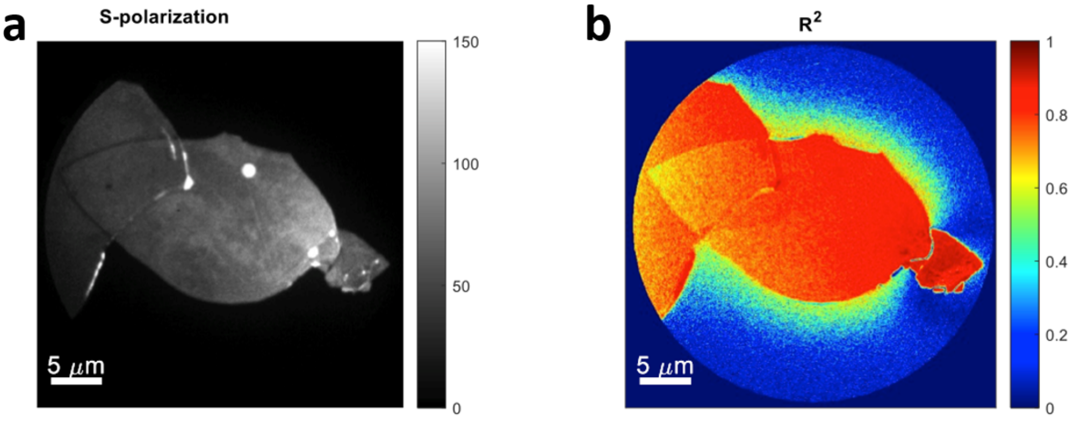

FIG. 6. PEEM images of flake 1 with 2.4 eV illumination. (a) S-polarized ($\theta = 0°$) PEEM image with correction for rhodium mirror reflectivity. (b) R^2 pixel-by-pixel map corresponding to β and ρ mapping shown in Figs. 2(a) and (b).

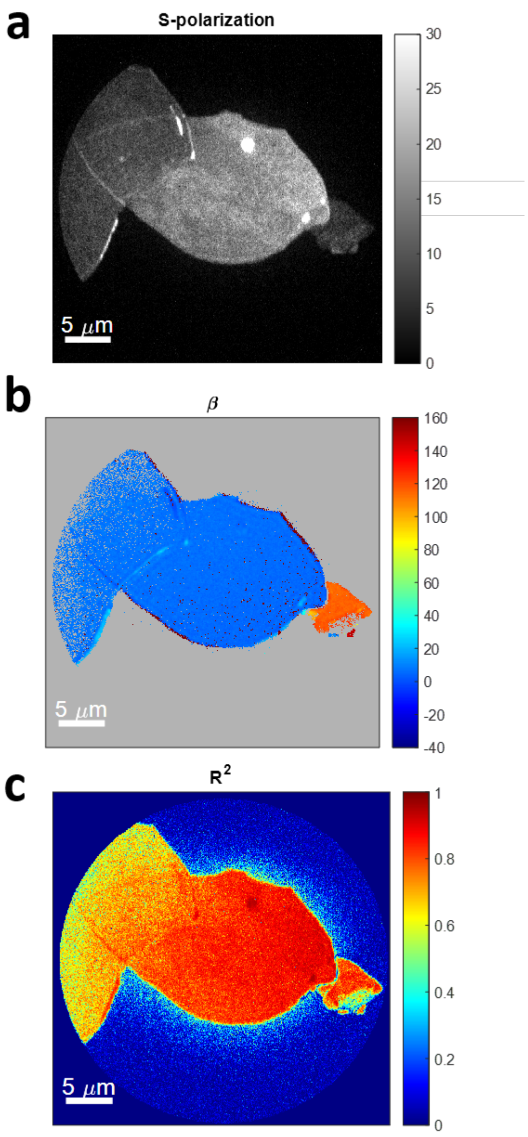

FIG. 7. PEEM images of flake 1 with 1.55 eV illumination. (a) S-polarized ($\theta = 0°$) PEEM image with correction for rhodium mirror reflectivity. (b) β map with goodness-of-fit thresholding for $R^2 > 0.6$. The behavior is qualitatively similar to that observed in Fig. 2(a). (c) R^2 pixel-by-pixel map corresponding to β and ρ mapping shown in (b) and Fig. 2(c).

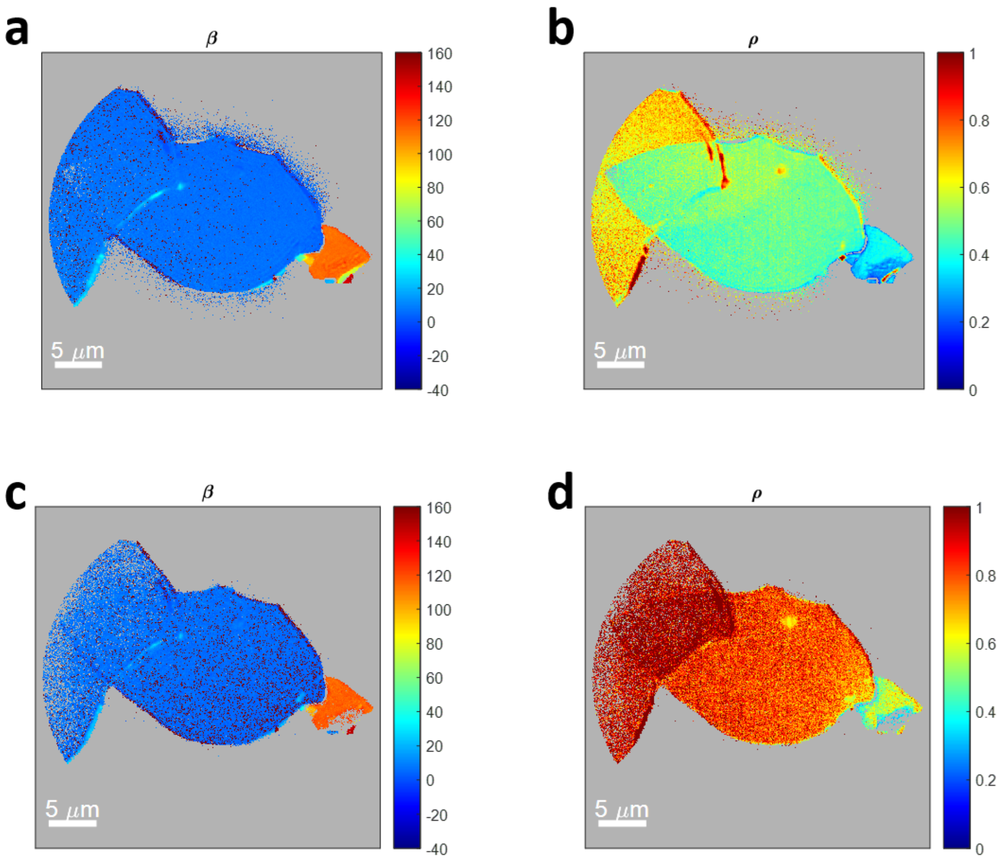

FIG. 8. Unfiltered β and ρ maps for 2.4 and 1.55 eV illumination. (a), (b) Unfiltered maps for hν=2.4 eV. (c), (d) Unfiltered maps for hν = 1.55 eV. All maps are shown with $R^2 > 0.6$ as shown in Figs. 2, 6, and 7.

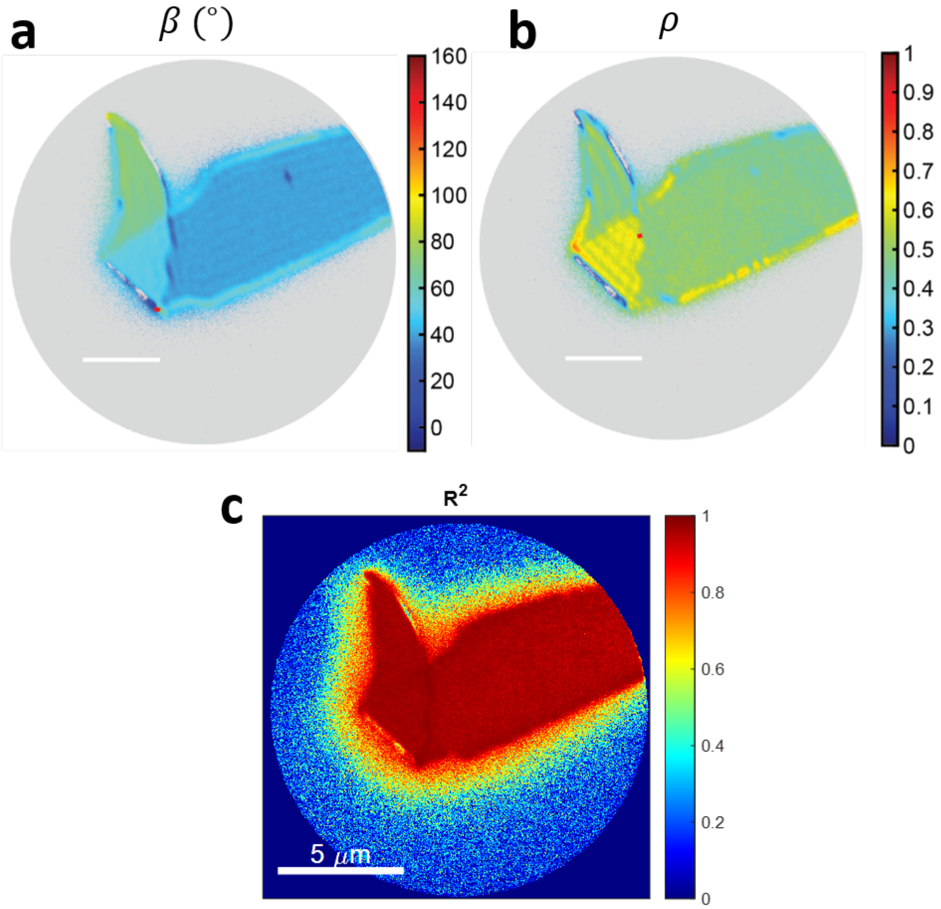

FIG. 9. Pixel-by-pixel mapping for flake 2 with 2.4 eV illumination. (a) β map, (b) ρ map. Both maps are shown with $R^2 > 0.8$. The locations of the β and ρ line cuts in Fig. 3 of the main paper are shown with small red lines. (c) R^2 goodness of fit map for (a) and (b).

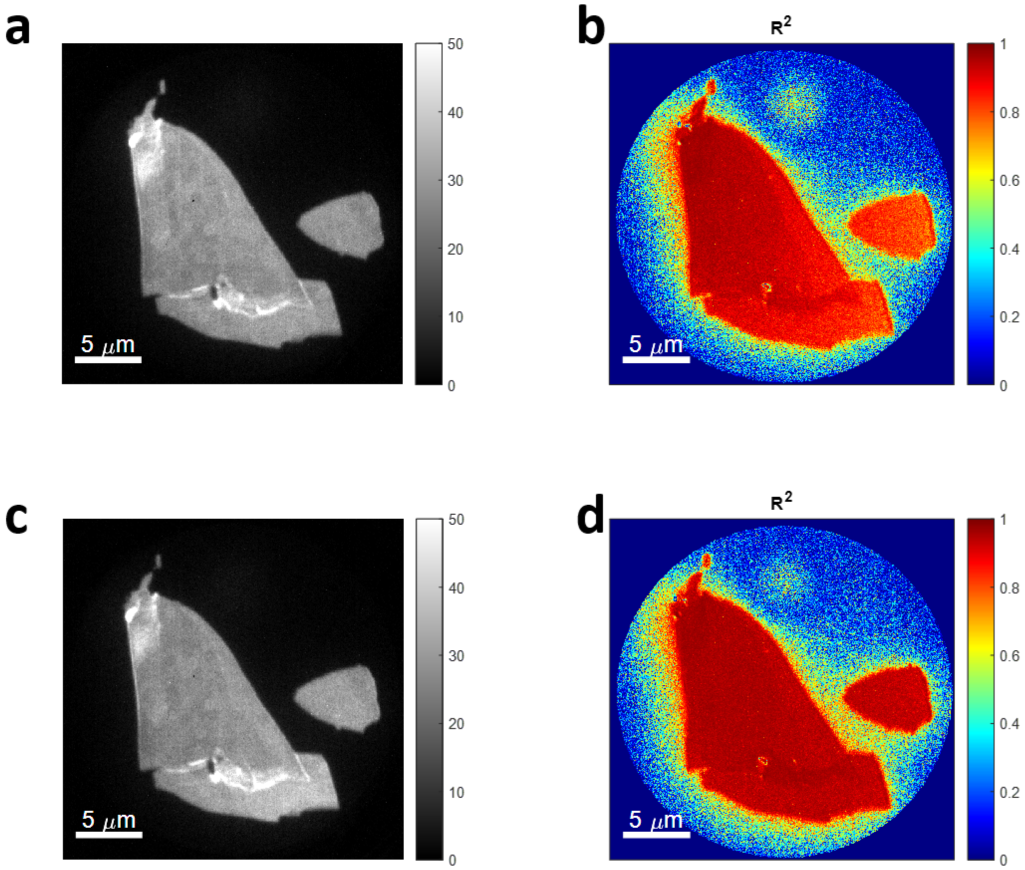

FIG. 10. PEEM images and R^2 maps for unannealed and annealed flake of Flake 3 with 2.4 eV illumination. (a) and (c) are the PEEM images taken at $\theta = 0°$ for the unannealed and 350°C annealed flake, respectively. (b) and (d) are the R^2 maps for the unannealed and 350°C annealed flake, respectively. Annealing marginally improves the photoemission signal and pixel-by-pixel fitting quality.

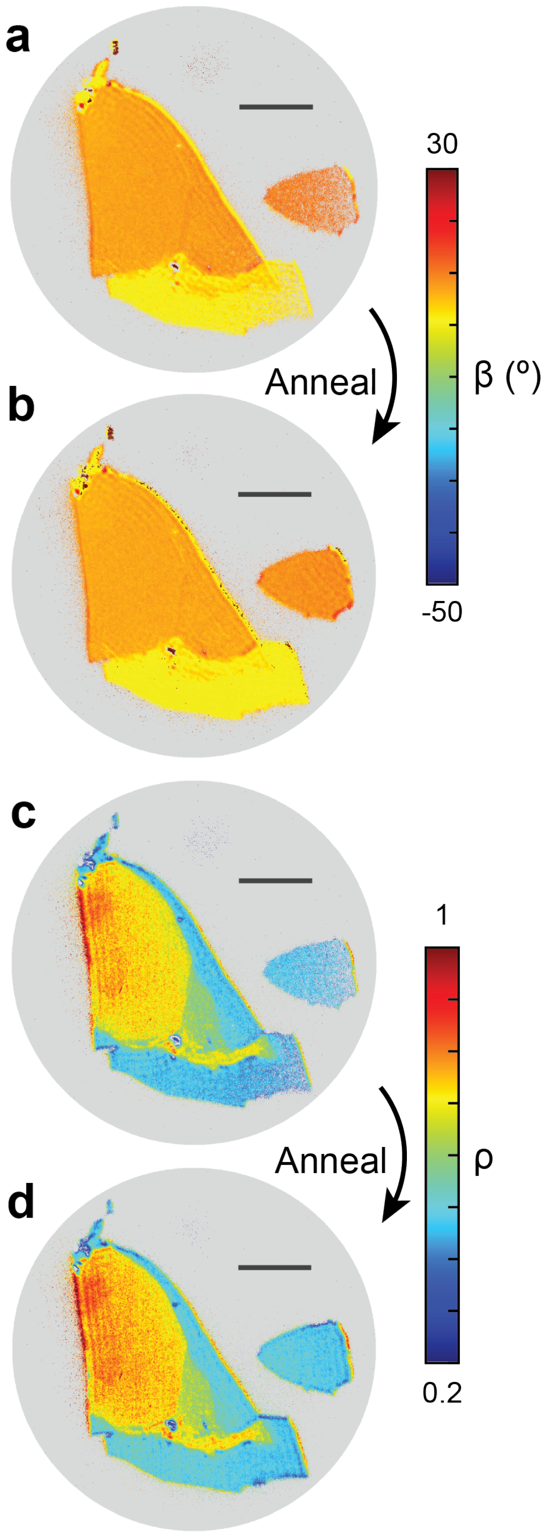

FIG. 11. (a) 2.4 eV β mapping of Flake 3 before and (b) after annealing at 350 °C. (c) 2.4 eV ρ mapping before and (d) after annealing at 350 °C. Scale bar: 5 μm. All maps are median-filtered and only pixels with an $R^2 > 0.8$ are shown.

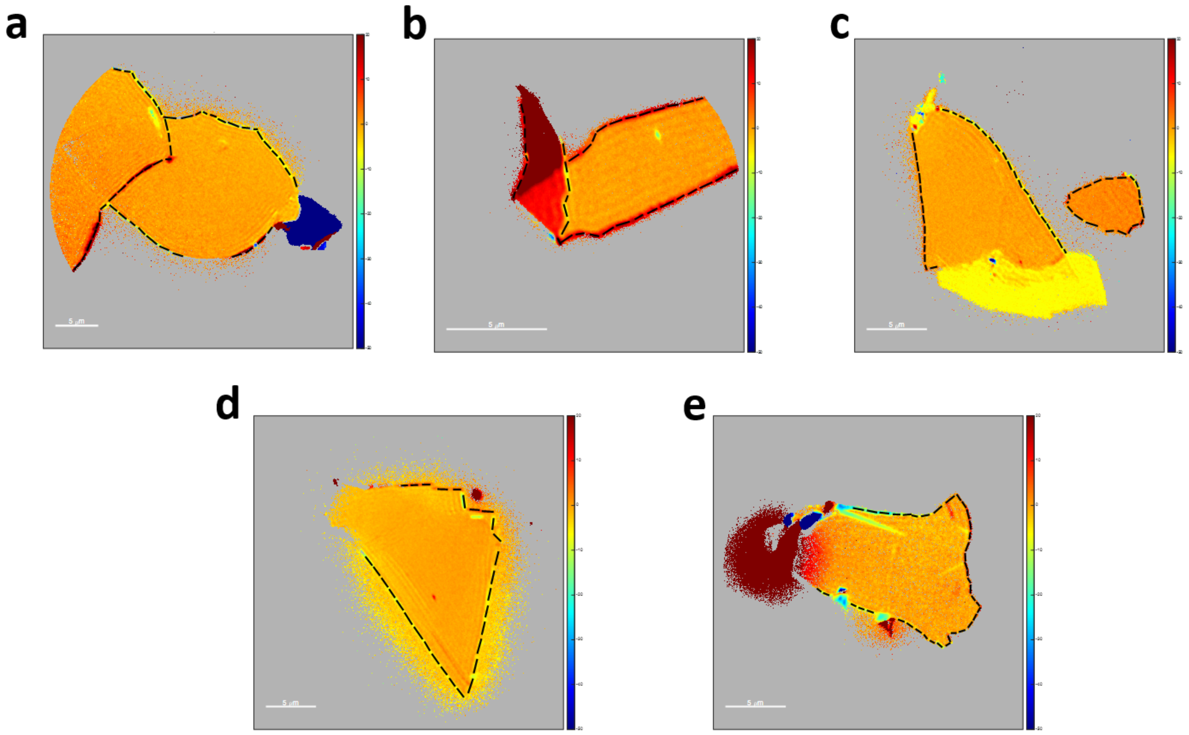

FIG. 12. $\delta\beta$ map linecuts of flake edges in Fig. 4. (a) Flake 1 (b) Flake 2 (c) Flake 3 (d) Flake 4 (e) Flake 5. R^2thresholds for (a) and (e) are 0.6, (b), (c), and (d) are 0.8. The β values for the flake interiors represent the rotation of the lattice armchair axis with respect to the lab frame in Fig. 1.They are as follows for these flakes: (a) Flake 1: 7°, (b) Flake 2: 40°, (c) Flake 3: 7°, (d) Flake 4: 38°, (e) Flake 5: 156°.

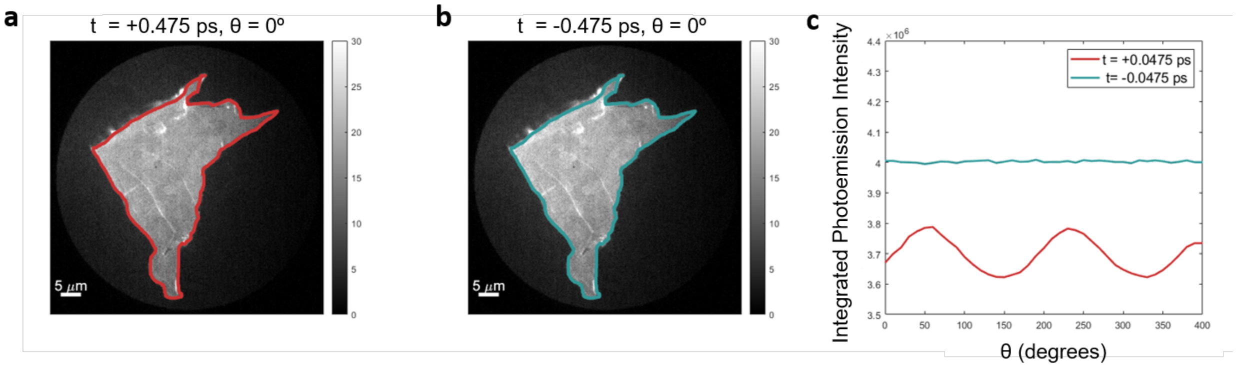

FIG. 13. Polarization dependence of hν=1.55 with 1.55 eV pump and 3.1 eV probe of flake 6. (a) Photoemission with $\theta = 0°$ where 1.55 eV pump arrives 0.475 before probe (+0.475 ps) (b) Photoemission with $\theta = 0°$ where 1.55 eV pump arrives 0.475 after probe (-0.475 ps) (c) Polarization dependence at +0.475 and -0.475 ps of the integrated areas shown in (a) and (b). The polarization response is clearly not due to the final state and is dependent on the absorption of the first photon

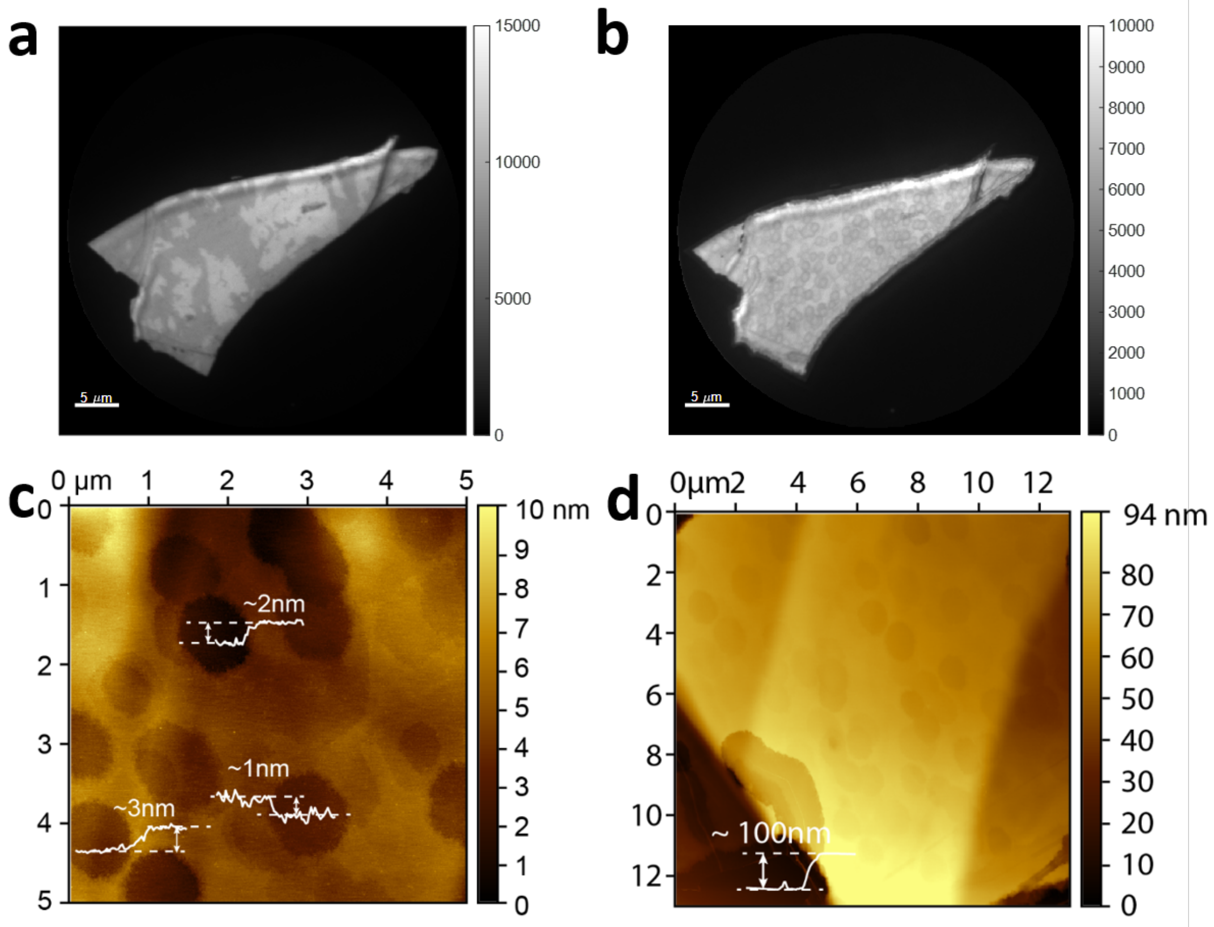

FIG. 14. Sublimation of BP flake 7. (a) PEEM image of BP flake taken with continuous-wave broadband Hg illumination before heating (b) PEEM image of BP flake with Hg illumination after heating to $> 400°C$ (c) AFM of holes with depth profiles of 1-3 nm (d) AFM of flake edge showing a thickness of ∼100 nm

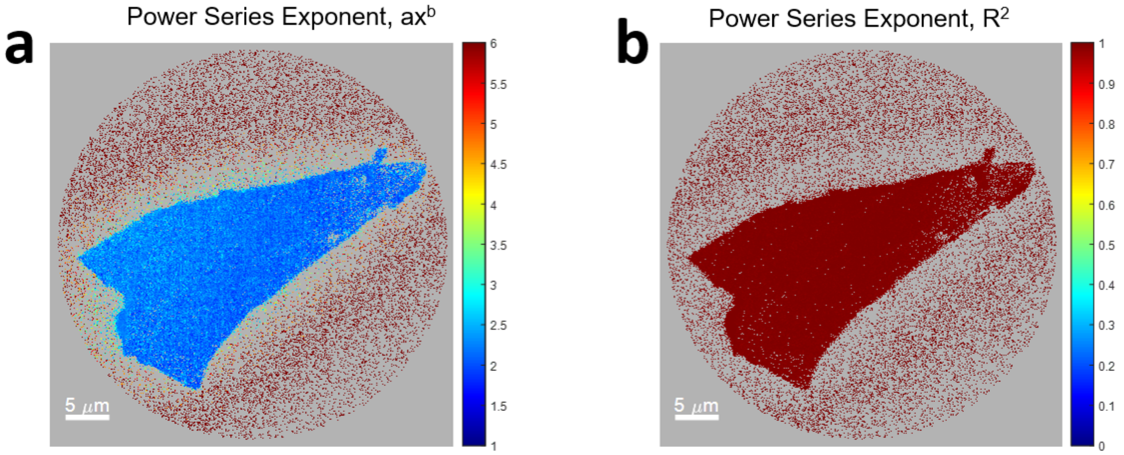

FIG. 15. Pixel-by-pixel power series map of unannealed BP flake 7 with $h\nu = 2.4$ eV excitation. Intensities are fit to Ax^b, where b indicates the number of photons required for photoemission. (a) Map of b for each pixel and (b) goodness of fit R^2. Maps are pixels that exceed the threshold $R^2 = 0.98$.

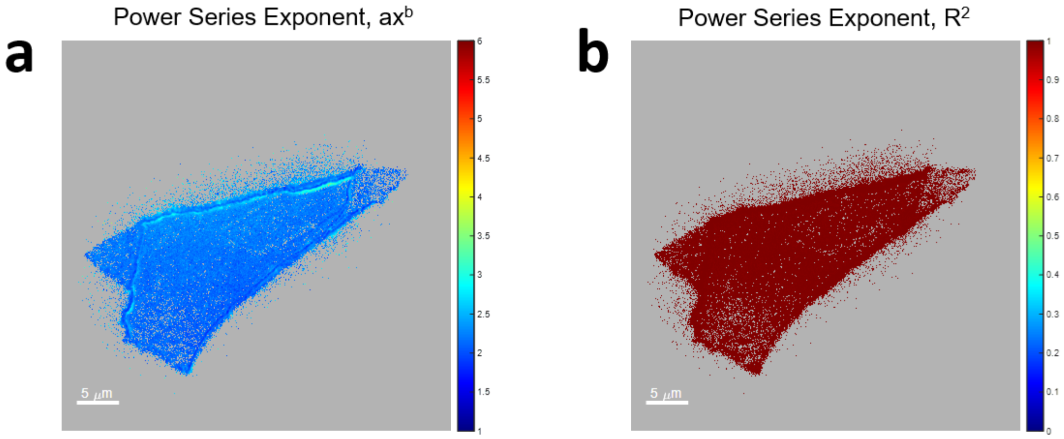

FIG. 16. Pixel-by-pixel power series map of sublimated BP flake 7 with $h\nu = 2.4$ eV excitation. Intensities are fit to Ax^b, where b indicates the number of photons required for photoemission. (a) map of b for each pixel and (b) goodness of fit R^2. Maps are pixels that exceed the threshold $R^2 = 0.99$.

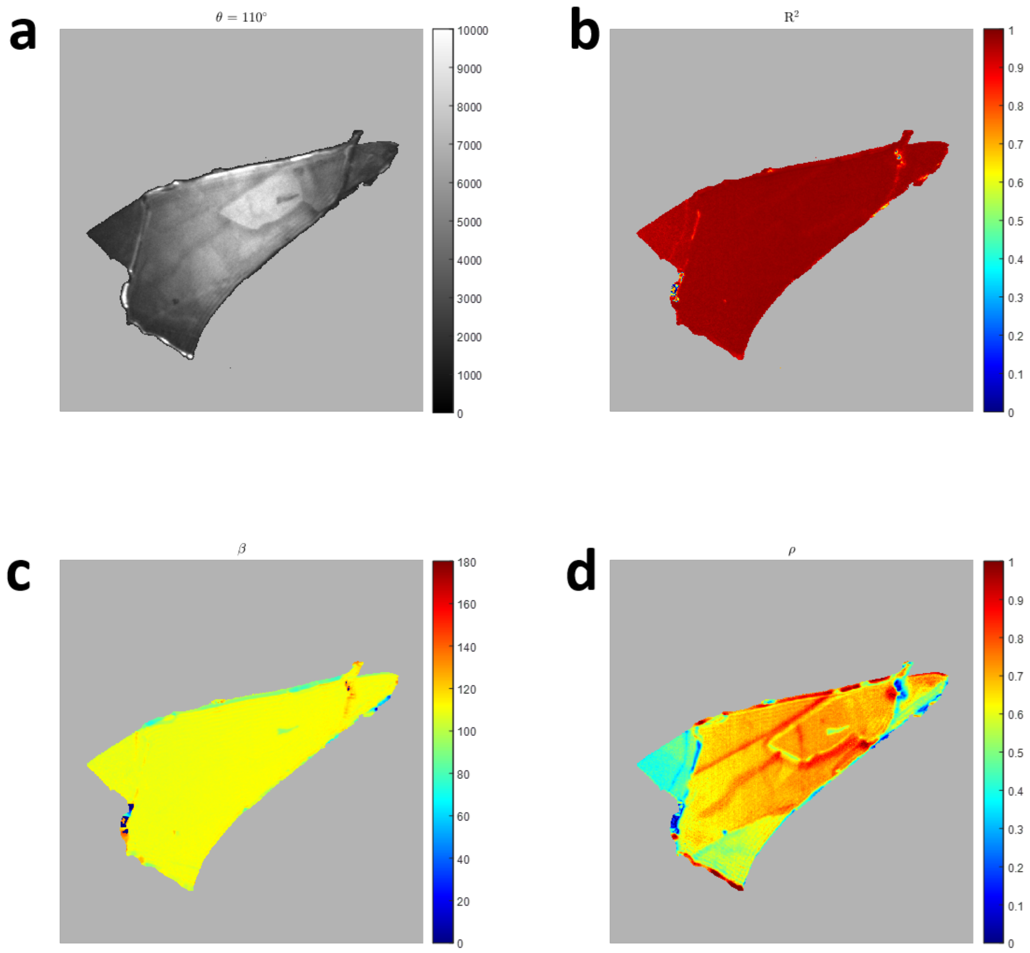

FIG. 17. Pixel-by-pixel polarization dependence map of unannealed BP flake with $h\nu = 2.4$ eV excitation. (a) PEEM image where $\theta = 110°$ and (b) goodness of fit R^2 map (c) β map, which shows that the AC direction is aligned approximately along $\theta = 110°$ (d) ρ map. All maps show pixels that correspond to those that exceed a photoemission intensity of 1200 counts.

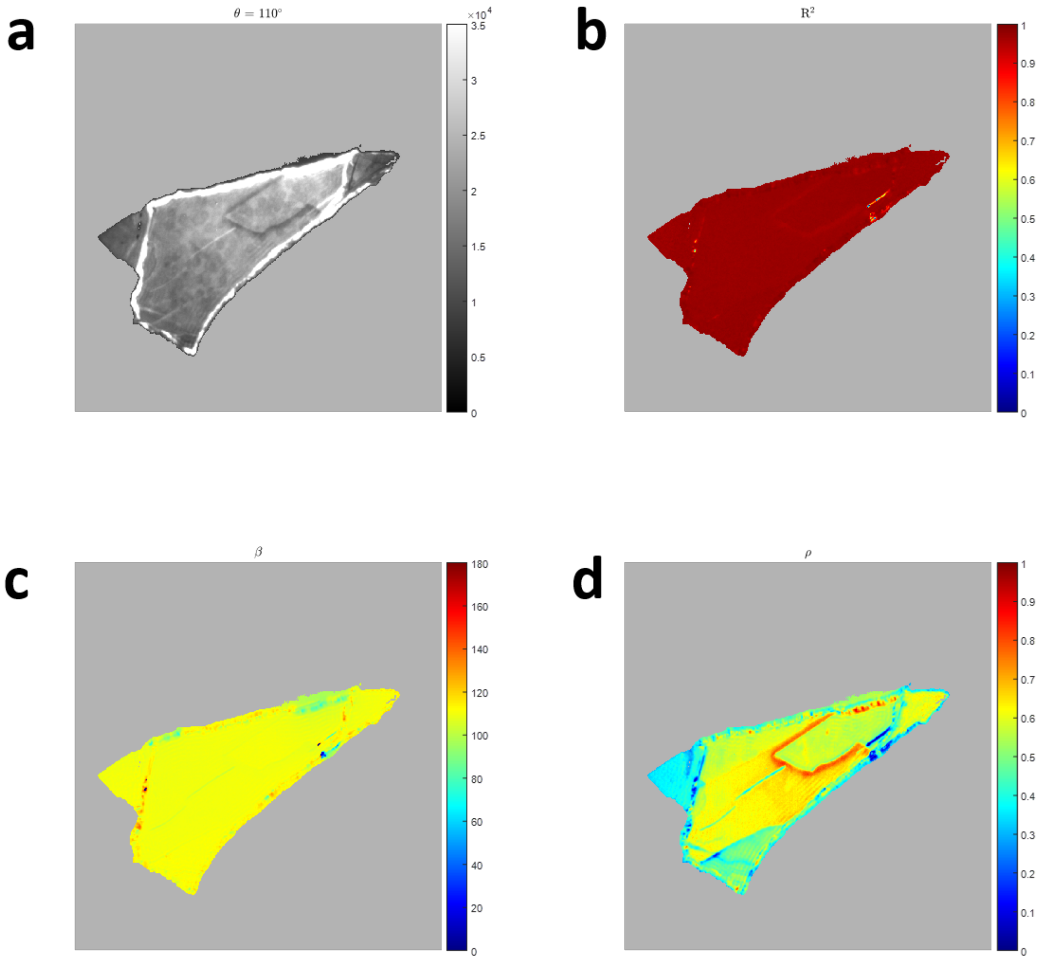

FIG. 18. Pixel-by-pixel polarization dependence map of sublimated BP flake with $h\nu = 2.4$ eV excitation. (a) PEEM image where $\theta = 110°$ (b) Goodness of fit R^2 map (c) β map, which shows that the AC direction is aligned approximately along $\theta = 110°$ (d) ρ map. All maps show pixels that correspond to those that exceed a photoemission intensity of 8000 counts.

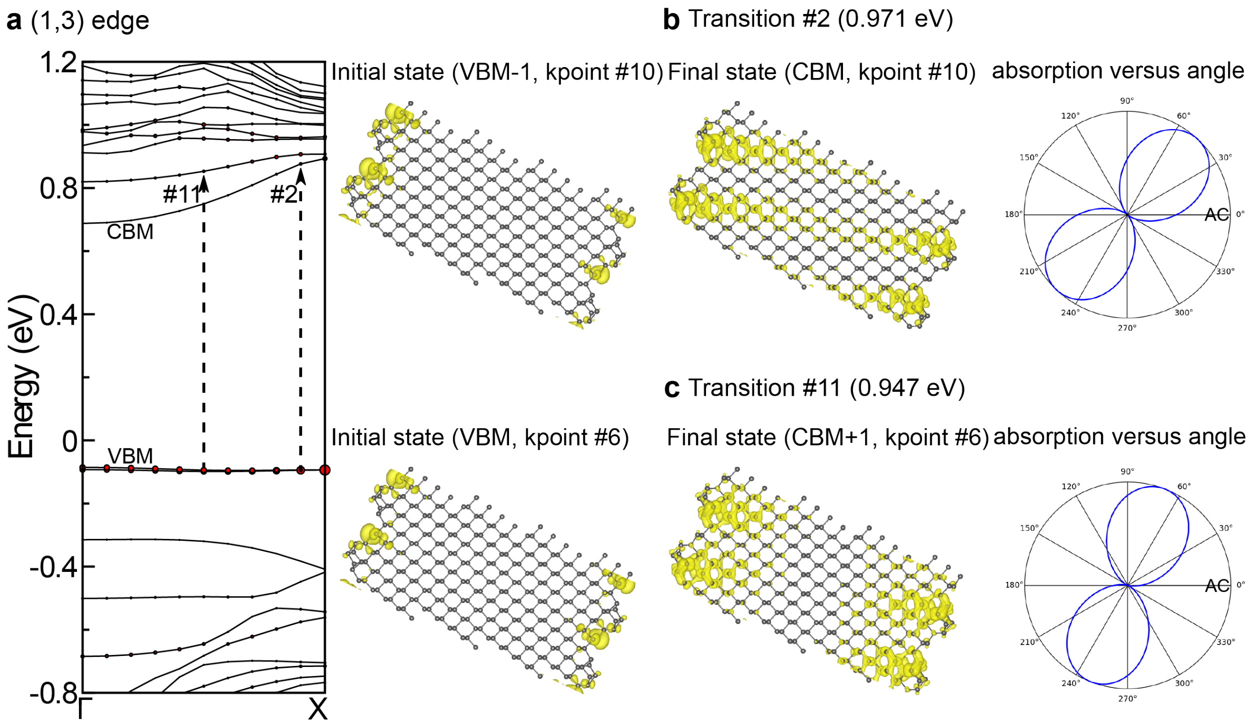

FIG. 19. (a) DFT calculated electronic band structure of the monolayer BP nanoribbon with a (1,3) reconstructed edge. The Fermi level is set at 0 eV. The valence band maximum and conduction band minimum are denoted as VBM and CBM, respectively. Red circles superimposed on the bands correspond to projected bands from the edge atoms. The size of an circle is proportional to the amplitude of the contribution of the edge atoms to a band state. The VBM and VBM-1 that are very close in energy are the only bands that have dominant contributions from the edge (i.e., edge bands). Two optical transitions around 0.95 eV are indicated by the arrows, and their charge density distributions of initial and final band states are shown in (b-c). For each transition, the polar profile of the absorption intensity versus the polarization angle is also presented. The armchair (AC) direction of BP is along the horizontal direction (i.e., 0.0°).

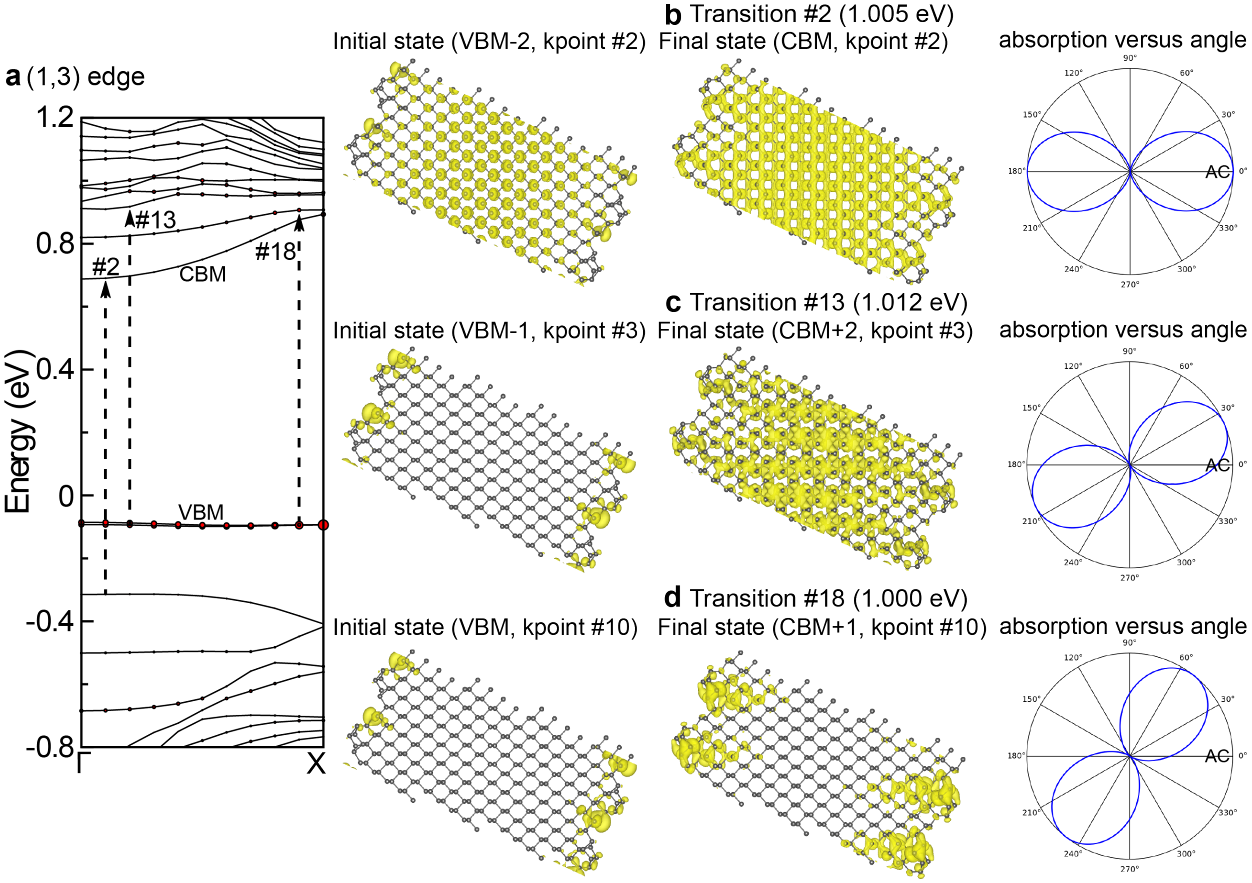

FIG. 20. (a) DFT calculated electronic band structure of the BP nanoribbon with the (1,3) edge. Red circles superimposed on the bands correspond to projected bands from the edge atoms. Three optical transitions at or near 1.0 eV are indicated by the arrows, and their charge density distributions of initial and final states are shown in (b-d). For each transition, the polar profile of the absorption intensity versus the polarization angle is also presented. The armchair (AC) direction of BP is along the horizontal direction (i.e., 0.0°).

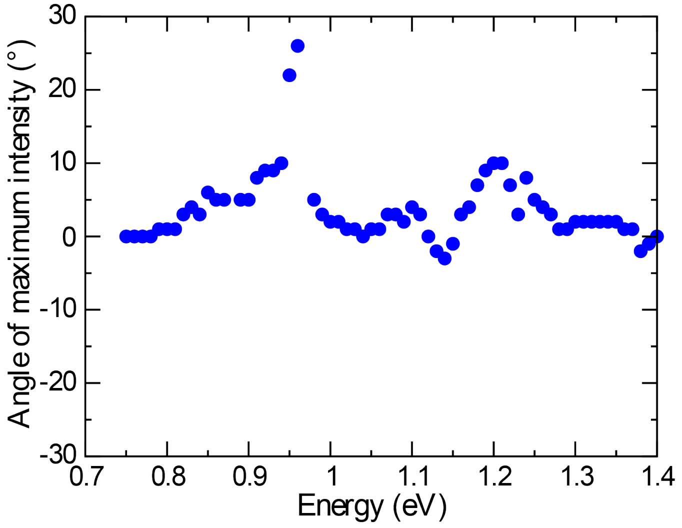

FIG. 21. DFT calculated averaged angle of maximum intensity as a function of the excitation energy for the BP nanoribbon with the (1,3) edge. The AC direction of BP is defined at 0°, so the angle of maximum intensity for the (1,3) edge is equivalent to the phase shift with respect to the AC direction. In the studied energy range, the averaged phase shift is mostly within ±10°.

Transition #	Energy (eV)	Band indices of initial and final states	kpoint #	Maximum intensity	Angle of maximum intensity (°)
1	0.939	349 $\rightarrow$ 351	9	8.69	57.0
2	0.971	349 $\rightarrow$ 351	10	13.40	47.0
3	0.927	349 $\rightarrow$ 352	4	27.37	5.0
4	0.938	349 $\rightarrow$ 352	5	24.55	8.0
5	0.951	349 $\rightarrow$ 352	6	21.22	12.0
6	0.966	349 $\rightarrow$ 352	7	17.52	15.0
7	0.938	350 $\rightarrow$ 351	9	22.97	8.0
8	0.970	350 $\rightarrow$ 351	10	10.96	13.0
9	0.921	350 $\rightarrow$ 352	4	4.91	73.0
10	0.933	350 $\rightarrow$ 352	5	6.29	72.0
11	0.947	350 $\rightarrow$ 352	6	6.97	69.0
12	0.963	350 $\rightarrow$ 352	7	7.48	65.0
13	0.979	350 $\rightarrow$ 352	8	8.58	60.0
average				11.73	22.0

TABLE I. For an optical transition of 0.95 eV in the monolayer BP nanoribbon with the (1,3) reconstructed edge, we considered all possible transitions between the valence and conduction bands (13 in total). For each transition, its energy, band indices of the valence and conduction states, and the kpoint index are shown. The indices of the valence band maximum (VBM) and conduction band minimum (CBM) are 350 and 351, respectively. For each transition, the transition dipole moment is calculated, which subsequently yields the polarization-angle-dependent optical absorption according to Eq. 3. The maximum intensity and the polarization angle corresponding to the maximum are shown in the table. The armchair (AC) direction of BP is at 0.0°. The averaged angle-dependent intensity is obtained by considering equal contributions from all the transitions, and its maximum value and the corresponding angle are listed at the table bottom. Two representative transitions (#2 and #11) as highlighted in light pink color are illustrated in Fig. 19.

Transition #	Energy (eV)	Band indices of i and f states	kpoint #	Maximum int.	Angle of maximum int. (°)
1	1.002	$348 \rightarrow 351$	1	386.26	0.0
2	1.005	$348 \rightarrow 351$	2	384.81	0.0
3	1.011	$348 \rightarrow 351$	3	380.35	0.0
4	1.023	$348 \rightarrow 351$	4	372.56	0.0
5	0.971	$349 \rightarrow 351$	10	13.40	47.0
6	0.987	$349 \rightarrow 351$	11	19.12	39.0
7	0.981	$349 \rightarrow 352$	8	13.22	19.0
8	0.994	$349 \rightarrow 352$	9	8.02	24.0
9	1.001	$349 \rightarrow 352$	10	2.83	37.0
10	1.001	$349 \rightarrow 352$	11	1.67	68.0
11	1.004	$349 \rightarrow 353$	1	0.004	53.0
12	1.003	$349 \rightarrow 353$	2	1.08	27.0
13	1.012	$349 \rightarrow 353$	3	1.29	23.0
14	1.027	$349 \rightarrow 354$	1	5.45	29.0
15	0.987	$350 \rightarrow 351$	11	4.22	33.0
16	0.979	$350 \rightarrow 352$	8	8.58	60.0
17	0.993	$350 \rightarrow 352$	9	10.61	55.0
18	1.000	$350 \rightarrow 352$	10	12.93	51.0
19	1.001	$350 \rightarrow 352$	11	11.99	54.0
20	0.997	$350 \rightarrow 353$	1	1.64	20.0
21	0.995	$350 \rightarrow 353$	2	0.81	10.0
22	1.005	$350 \rightarrow 353$	3	0.28	167.0
23	1.029	$350 \rightarrow 353$	4	0.09	118.0
24	1.020	$350 \rightarrow 354$	1	0.01	30.0
average				66.29	2.0

TABLE II. Possible optical transitions with 1.0 eV in the BP nanoribbon with the (1,3) edge. For each transition, its energy, band indices of the valence and conduction states, the kpoint index, the maximum intensity, and the polarization angle corresponding to the maximum are shown were 0.0° is the AC direction. The averaged angle-dependent intensity is considers equal contributions from all the transitions, and the corresponding intensity and corresponding angle are listed on the bottom. Highlighted transitions are shown in Fig. 20.
